# Supplementary material for: Molecular mechanism of phospholipid transport at the bacterial outer membrane interface
Source: Nat Commun. 2023 Dec 13;14:8285. doi: 10.1038/s41467-023-44144-8 (PMC10719372; doi:10.1038/s41467-023-44144-8)
Supplement: Supplementary file 1 — Supplementary Information [file 41467_2023_44144_MOESM1_ESM.pdf]

Supplementary Information

Molecular mechanism of phospholipid transport at the bacterial outer membrane interface

Jiang Yeow[1], Min Luo[2], and Shu-Sin Chng[1,3,*]

Affiliation:

[1]Department of Chemistry, Faculty of Science, National University of Singapore, Singapore 117543

[2]Department of Biological Sciences, Faculty of Science, National University of Singapore, Singapore 117558

[3]Singapore Center for Environmental Life Sciences Engineering, National University of Singapore (SCELSE-NUS), Singapore 117456

[*]To whom correspondence should be addressed: chmchngs@nus.edu.sg

This PDF file includes:

Supplementary Figures 1 to 13

Supplementary Tables 1 to 5

Supplementary References

Supplemental Figures

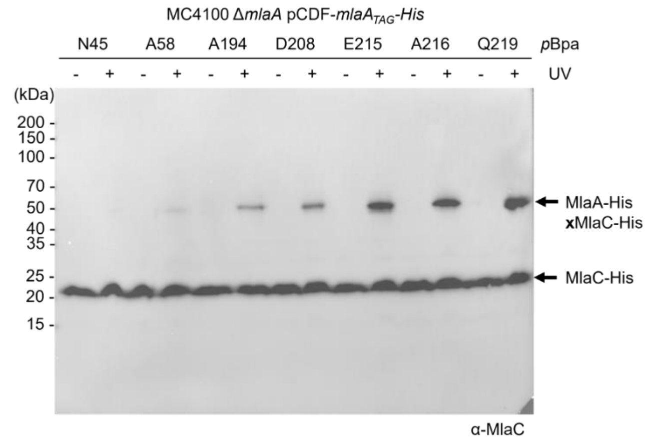

Supplementary Figure 1. Five more positions on the C-terminal α-helices of MlaA at the base of the hydrophilic channel interact with MlaC. Representative immunoblots showing UV-dependent formation of weak crosslinks between MlaA and MlaC in Δ*mlaA* cells expressing MlaA substituted with *p*Bpa at indicated positions from the pCDF plasmid. The experiment had been performed at least three times with similar results. Source data are provided as a Source Data file.

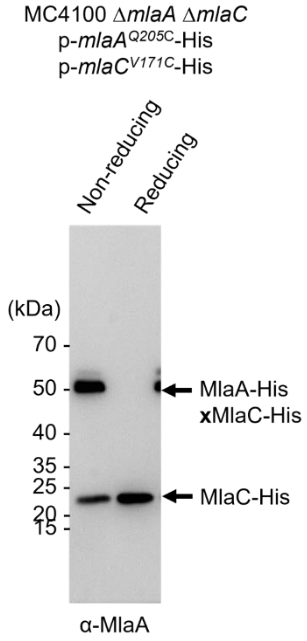

Supplementary Figure 2. Residue V171 at the opening of the lipid binding cavity of MlaC forms disulfide bonds extensively with residue Q205 at the periplasmic base of MlaA. Representative immunoblots showing formation of disulfide crosslinks between MlaA^{Q205C} and MlaC^{V171C} in Δ*mlaA* Δ*mlaC* cells expressing cysteine-substituted MlaA-His and MlaC-His from the pCDF and pET22/42 plasmids, respectively. The crosslinked band can be detected by α-MlaA (here) and α-MlaC (Fig. 2A). Samples were subjected to non-reducing or reducing SDS-PAGE prior to immunoblotting. The experiment had been performed at least three times with similar results. Source data are provided as a Source Data file.

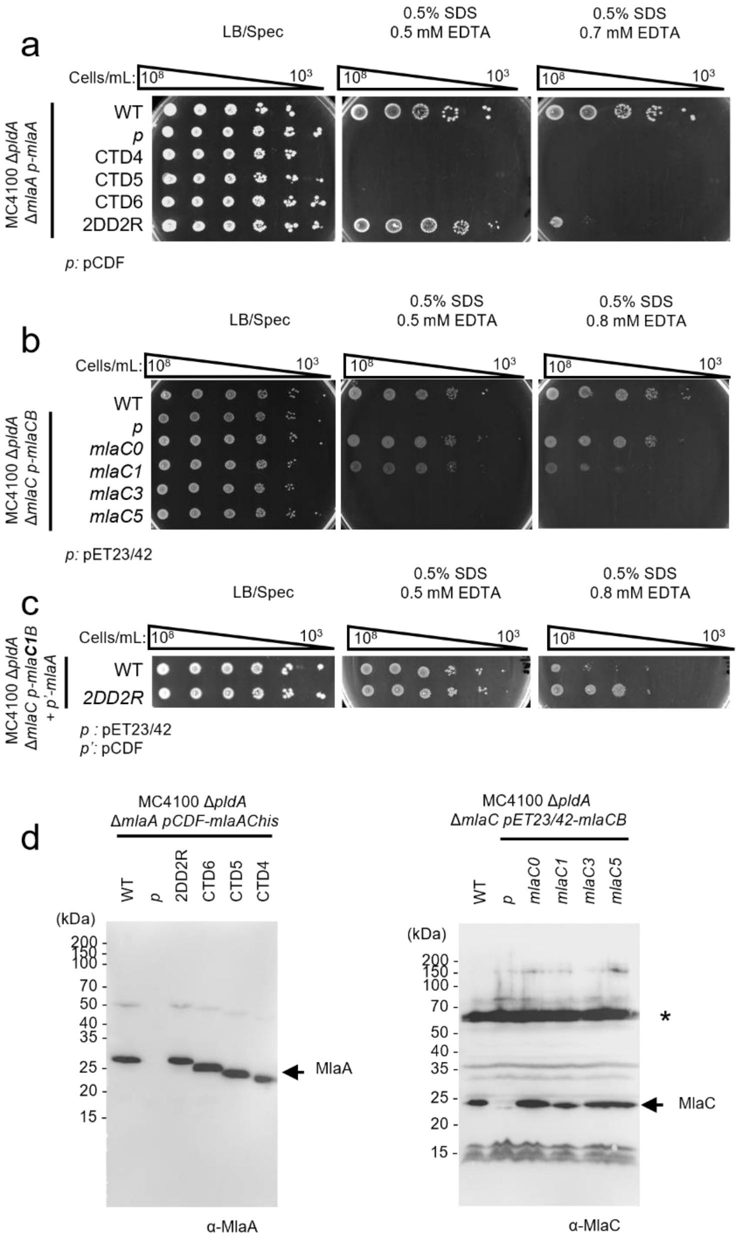

Supplementary Figure 3. Deletion of C-terminal tail helix and charge-reversals of MlaA, as well as charge-reversals at surface charge patches on MlaC, perturb Mla function in Δ*pldA* Δ*mla* strains. Analyses of SDS/EDTA sensitivity of (A) Δ*pldA* Δ*mlaA* strains producing indicated MlaA C-terminal tail helix deletion and charge-reversed mutants from the pCDF vector, (B) Δ*pldA* Δ*mlaC* strains producing indicated MlaC surface charge-reversed mutant variants from the pET23/42 vector, and (C) Δ*pldA* Δ*mlaC* strains co-expressing both indicated MlaC and MlaA variants. Mutations in MlaA and MlaC do not significantly affect protein expression levels in cells, as shown in corresponding immunoblots in (D). The experiment had been performed at least three times with similar results. Source data are provided as a Source Data file.

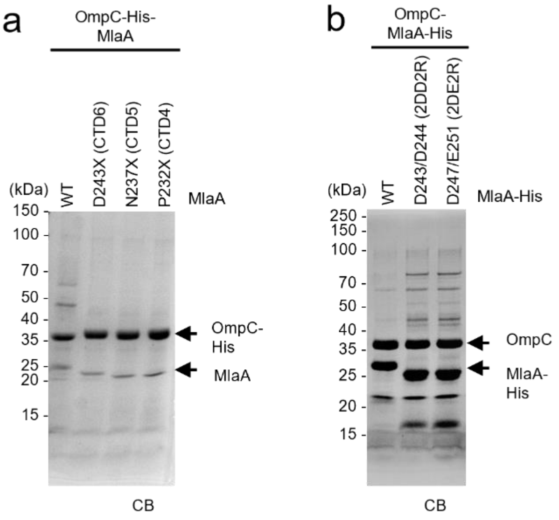

Supplementary Figure 4. MlaA C-terminal tail helix mutations do not affect OmpC-MlaA complex formation. Purified (A) (OmpC-His)-MlaA with indicated MlaA C-terminal tail helix deletion variants, and (B) OmpC-(MlaA-His) with indicated MlaA C-terminal tail helix charge-reversed variants subjected to SDS-PAGE, followed by Coomassie blue (CB) staining. The experiment had been performed at least three times with similar results. Source data are provided as a Source Data file.

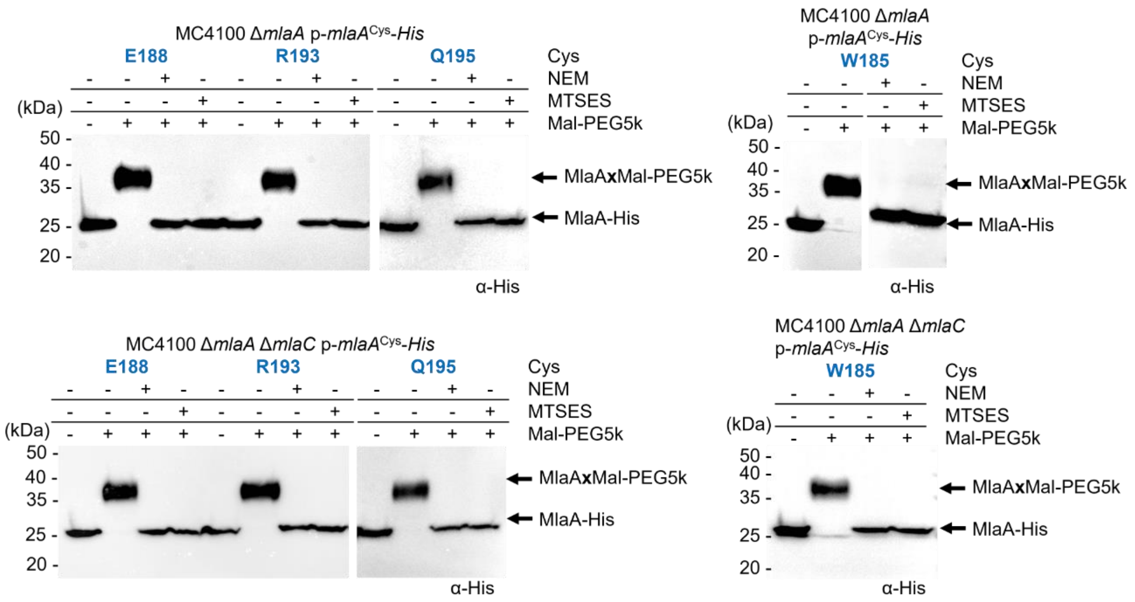

Supplementary Figure 5. Other selected MlaA channel residues do not display solvent accessibility changes in the absence of MlaC. Representative immunoblots showing Mal-PEG5k alkylation of MlaA variants containing channel residues substituted with cysteine (in Δ*mlaA* or Δ*mlaA* Δ*mlaC* strains) following labelling by membrane permeable N-ethylmaleimide (NEM) or impermeable (MTSES) reagents. Mal-PEG5k alkylated MlaA^{Cys}-His variants show an approximate ~5 kDa mass shift. Positions fully blocked by MTSES, which reflects the level of solvent accessibility, are highlighted in *blue*. These experiments had been performed at least three times with similar results. Source data are provided as a Source Data file.

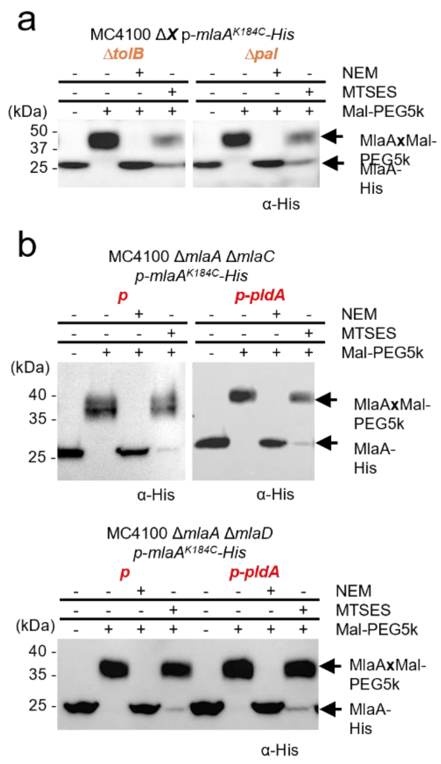

Supplementary Figure 6. MlaA channel solvent accessibility changes at residue K184 is not influenced by perturbation of OM lipid asymmetry. Representative immunoblots showing Mal-PEG5k alkylation of MlaA^{K184C}-His variant expressed from the pCDF plasmid, either in **(A)** background strains harboring disrupted lipid asymmetry (i.e. Δ*tolB* and Δ*pal*) or **(B)** in Δ*mlaA* Δ*mlaC* or Δ*mlaA* Δ*mlaD* strains also overproducing PldA from the pBR322 plasmid. Cells were labelled with membrane-permeable (NEM) or impermeable (MTSES) reagents, followed by alkylation with Mal-PEG5k, which introduces a ~5-kDa mass shift to MlaA^{K184C}-His. The levels of solvent accessibility of K184C in MlaA in the various strains, i.e. partially, or not blocked by MTSES, are highlighted in *orange* or *red*, respectively. These experiments had been performed at least three times with similar results. Source data are provided as a Source Data file.

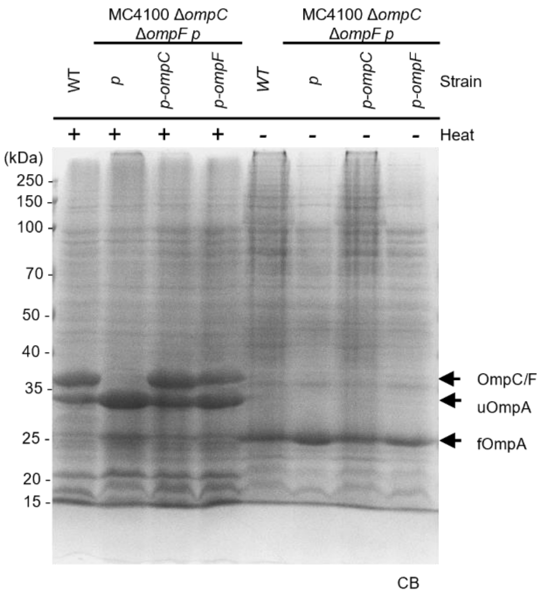

Supplementary Figure 7. OmpC and OmpF expressed in trans from the pDSW206 plasmid achieve native levels of porin expression (OmpC/F) in wild-type cells. Membrane fractions of wild-type or Δ*ompC* Δ*ompF* cells expressing OmpC or OmpF from pDSW206 plasmids were subjected to SDS-PAGE, followed by Coomassie blue (CB) staining. The experiment had been performed at least three times with similar results. Source data are provided as a Source Data file.

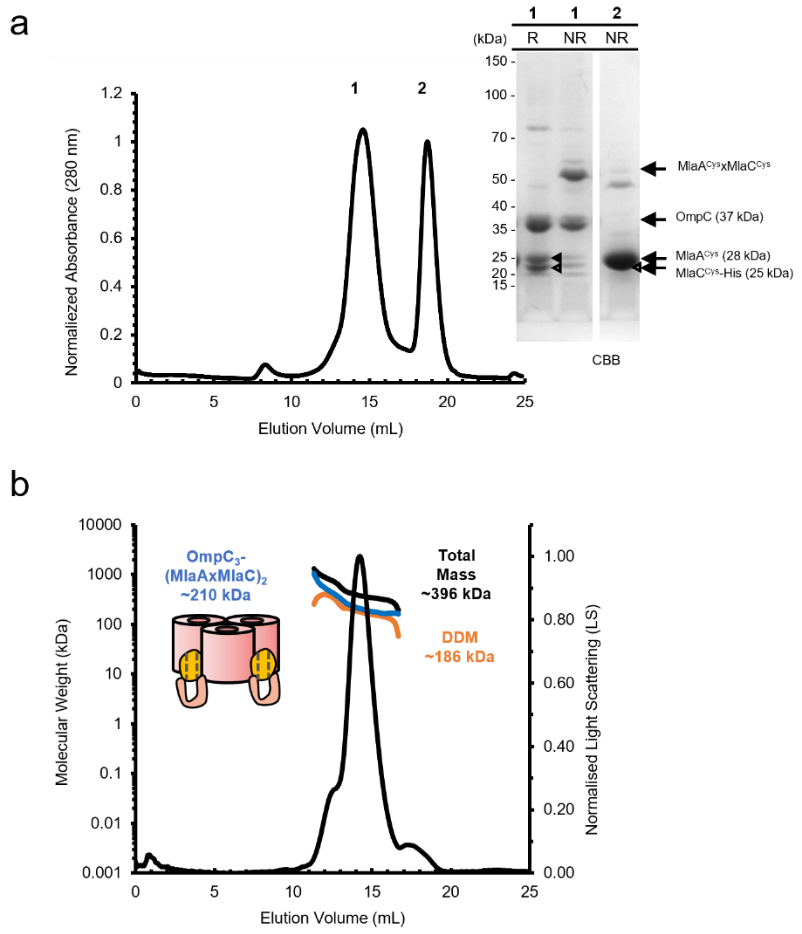

Supplementary Figure 8. The disulfide-trapped $OmpC_3$-MlaA^{Q205C}-MlaC^{V171C} complexes can be purified to homogeneity. **(A)** Size exclusion chromatographic (SEC) analysis of $OmpC_3$-MlaA^{Cys}-MlaC^{Cys}-His in DDM. Peaks 1 and 2 correspond to $OmpC_3$-MlaA^{Cys}-MlaC^{Cys}-His and free MlaC^{Cys}-His, respectively. Comparative reducing (R) and non-reducing (NR) SDS-PAGE analyses of peak fractions (*right*) revealed the formation of disulfide crosslink between MlaA^{Q205C} and MlaC^{V171C} in our purified preparation. These experiments had been performed at least three times with similar results. Source data are provided as a Source Data file. **(B)** SEC/multi-angle light scattering (MALS) analysis of $OmpC_3$-MlaA^{Cys}-MlaC^{Cys}-His in DDM. Molecular weight of $OmpC_3$-(MlaA^{Cys}-MlaC^{Cys})$_2$: ~213 kDa (predicted), ~210 (±2.2%) kDa (observed). Molecular weight of DDM fraction in the complex was ~186 (±5.7%) kDa. Numbers stated after ± show statistical consistency of analysis.

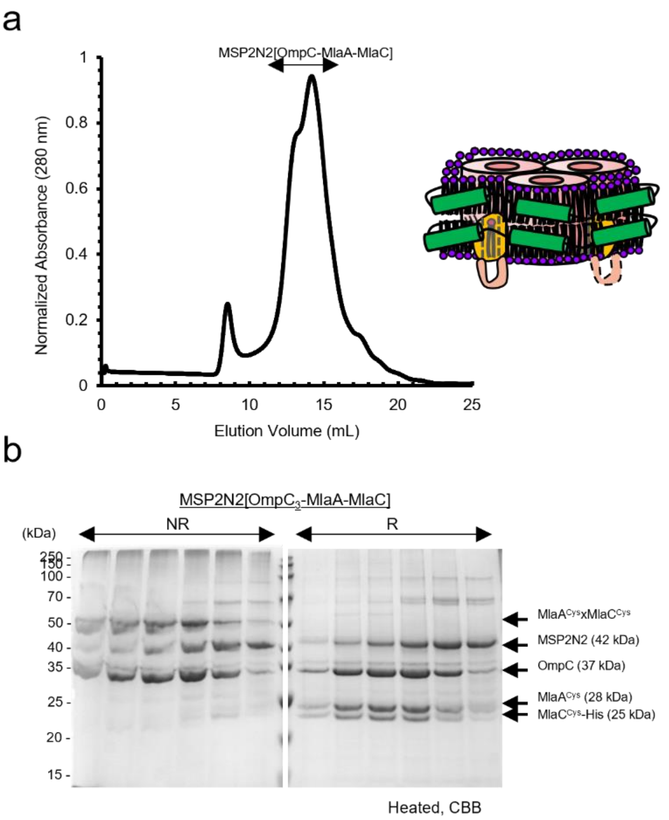

Supplementary Figure 9. Reconstitution of the OmpC3-MlaA^{Q205C}-MlaC^{V171C} complexes in nanodiscs. **(A)** SEC analysis of OmpC3-MlaA^{Cys}-MlaC^{Cys}-His reconstituted in MSP2N2 nanodiscs. **(B)** Peak fractions of interest were subjected to reducing (R) and non-reducing (NR) SDS-PAGE analyses. The experiment had been performed at least three times with similar results. Source data are provided as a Source Data file.

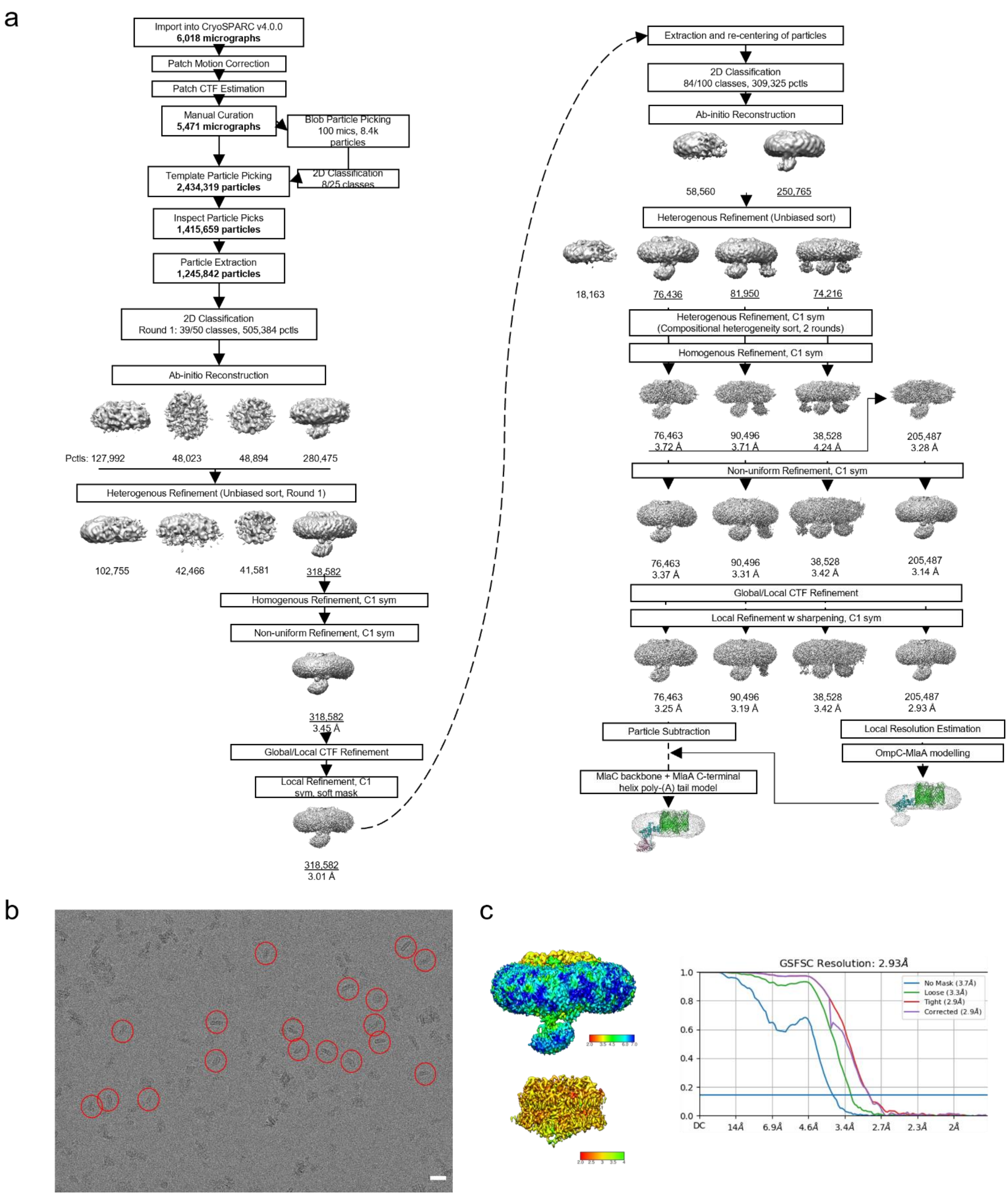
a
Import into CryoSPARC v4.0.0
6,018 micrographs
Patch Motion Correction
Patch CTF Estimation
Manual Curation
5,471 micrographs
Blob Particle Picking
100 mics, 8.4k particles
2D Classification
8/25 classes
Template Particle Picking
2,434,319 particles
Inspect Particle Picks
1,415,659 particles
Particle Extraction
1,245,842 particles
2D Classification
Round 1: 39/50 classes, 505,384 pctls
Ab-initio Reconstruction
Pctls: 127,992
48,023
48,894
280,475
Heterogenous Refinement (Unbiased sort, Round 1)
102,755
42,466
41,581
318,582
Homogenous Refinement, C1 sym
Non-uniform Refinement, C1 sym
318,582
3.45 Å
Global/Local CTF Refinement
Local Refinement, C1 sym, soft mask
318,582
3.01 Å
Extraction and re-centering of particles
2D Classification
84/100 classes, 309,325 pctls
Ab-initio Reconstruction
58,560
250,765
Heterogenous Refinement (Unbiased sort)
18,163
76,436
81,950
74,216
Heterogenous Refinement, C1 sym
(Compositional heterogeneity sort, 2 rounds)
Homogenous Refinement, C1 sym
76,463
3.72 Å
90,496
3.71 Å
38,528
4.24 Å
205,487
3.28 Å
Non-uniform Refinement, C1 sym
76,463
3.37 Å
90,496
3.31 Å
38,528
3.42 Å
205,487
3.14 Å
Global/Local CTF Refinement
Local Refinement w sharpening, C1 sym
76,463
3.25 Å
90,496
3.19 Å
38,528
3.42 Å
205,487
2.93 Å
Particle Subtraction
Local Resolution Estimation
OmpC-MlaA modelling
MlaC backbone + MlaA C-terminal helix poly-(A) tail model
b
c
GSFSC Resolution: 2.93Å
No Mask (3.7Å)
Loose (3.3Å)
Tight (2.9Å)
Corrected (2.9Å)
1.0
0.8
0.6
0.4
0.2
0.0
DC
14Å
6.9Å
4.6Å
3.4Å
2.7Å
2.3Å
2Å

Supplementary Figure 10. Single-particle cryo-EM analysis of nanodisc-embedded $OmpC_3$- $MlaA^{Q205C}$-$MlaC^{V171C}$ complexes. **(A)** Data processing flowchart yielding the final maps (**EMD-35321/2/3**) of three major classes representing compositional heterogeneity in $OmpC_3$-$(MlaA\text{-}MlaC)_x$. A fourth map for $OmpC_3$-$(MlaA\text{-}MlaC)_{1\text{-}3}$ (**EMD-35250**) was generated, where all particles from the above classes were combined and refined. The structure for $OmpC_3$-MlaA (**PDB 8I8R**) was built and refined in **EMD-35250**, while MlaC and the MlaA C-terminal tail helix (**PDB 8I8X**) were modelled in **EMD-35253**. Additional refinement details can be found in **Table S4**. **(B)** Representative cryo-EM image with several particles marked by circles. **(C)** Front and side orientations of the density map of $OmpC_3$-$(MlaA\text{-}MlaC)_{1\text{-}3}$ (**EMD-35250**) (unsharpened; contour level of 0.06, *transparency 80%*) with the protein surface densities colored *green* (OmpC; contour level of 0.1) and *cyan* (MlaA; contour level of 0.1) according to **PDB 8I8R**. Unsharpened and sharpened local resolution maps were calculated by cryoSPARC [1], and illustrated with pseudo-color representation of per-voxel resolution. The gold standard-Fourier Shell Correlation (GS-FSC) plots of unmasked and masked (loose, tight, and corrected) maps are derived from cryoSPARC from local refinement. Illustrations were generated using the software UCSF Chimera [2].

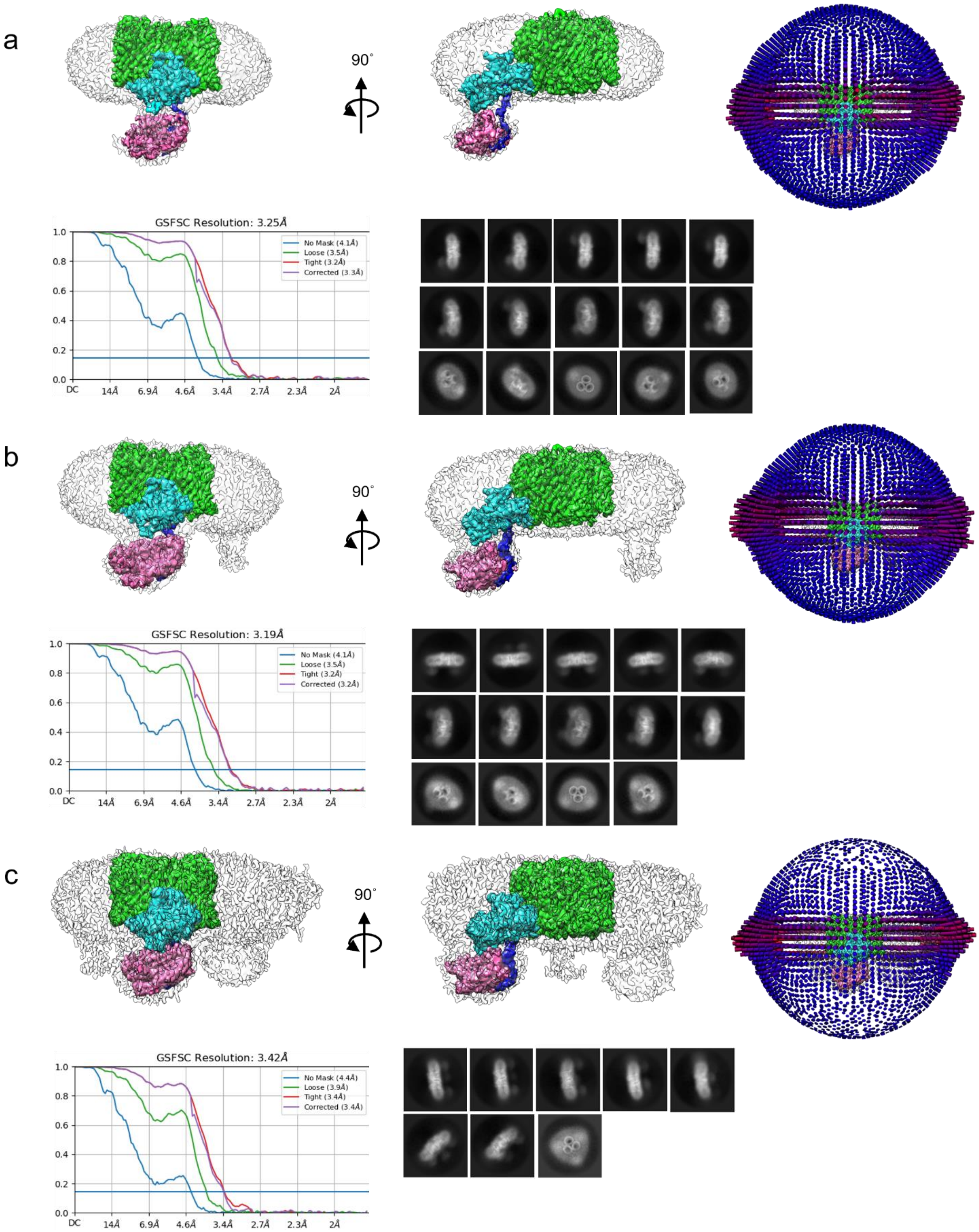
a
90°
GSFSC Resolution: 3.25Å
No Mask (4.1Å)
Loose (3.5Å)
Tight (3.2Å)
Corrected (3.3Å)
b
90°
GSFSC Resolution: 3.19Å
No Mask (4.1Å)
Loose (3.5Å)
Tight (3.2Å)
Corrected (3.2Å)
c
90°
GSFSC Resolution: 3.42Å
No Mask (4.4Å)
Loose (3.9Å)
Tight (3.4Å)
Corrected (3.4Å)
DC 14Å 6.9Å 4.6Å 3.4Å 2.7Å 2.3Å 2Å

Supplementary Figure 11. Cryo-EM density maps of various compositional variants of OmpC$_3$-(MlaA^{Q205C}-MlaC^{V171C})$_x$ in nanodiscs. **(A-C)** Relevant maps (sharpened/unsharpened) and parameters (Euler angle distributions, GS-FSC plots, representative 2D classes) for **(A)** OmpC$_3$-(MlaA-MlaC) (**EMD-35253**), **(B)** OmpC$_3$-(MlaA-MlaC)$_2$ (**EMD-35252**) and **(C)** OmpC$_3$-(MlaA-MlaC)$_3$ (**EMD-35251**) are presented. Sharpened (contour levels of 0.11, coloured *gray*) and unsharpened (contour levels of 0.06, coloured *white*, *transparency 80%*) densities are shown in *front* and *top* orientations. For Euler angle distributions, the height and color of each rod is proportional to the amount of particles visualized from the same specific orientation. The gold standard-Fourier Shell Correlation (GS-FSC) plots of unmasked and masked (loose, tight, and corrected) maps are derived from cryoSPARC. Representative 2D classes are generated using a particle box size of 320 pixels (275 Å). Illustrations were generated using the software UCSF Chimera [2].

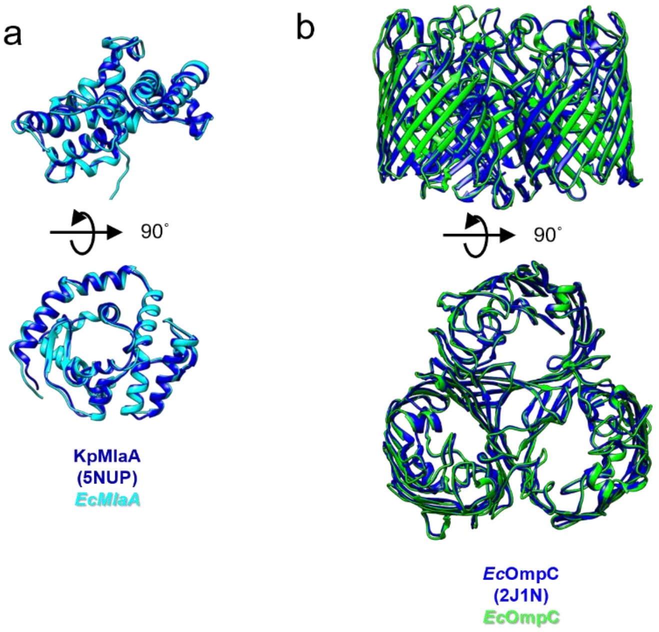

Supplementary Figure 12. Refined models of OmpC trimer and MlaA are highly similar to reported structures. Superimpositions of **(A)** OmpC trimer in our model (**PDB 8I8R**) with the reported OmpC trimer crystal structure (PDB 2J1N) [https://doi.org/10.2210/pdb2J1N/pdb], and of **(B)** *Ec*MlaA (**PDB 8I8R**) and the reported *Kp*MlaA structure in OmpK36-*Kp*MlaA (PDB 5NUP) [https://doi.org/10.2210/pdb5NUP/pdb] reveal insignificant root mean square deviations (r.m.s.d. <1 Å). Illustrations were generated using the software UCSF Chimera [2].

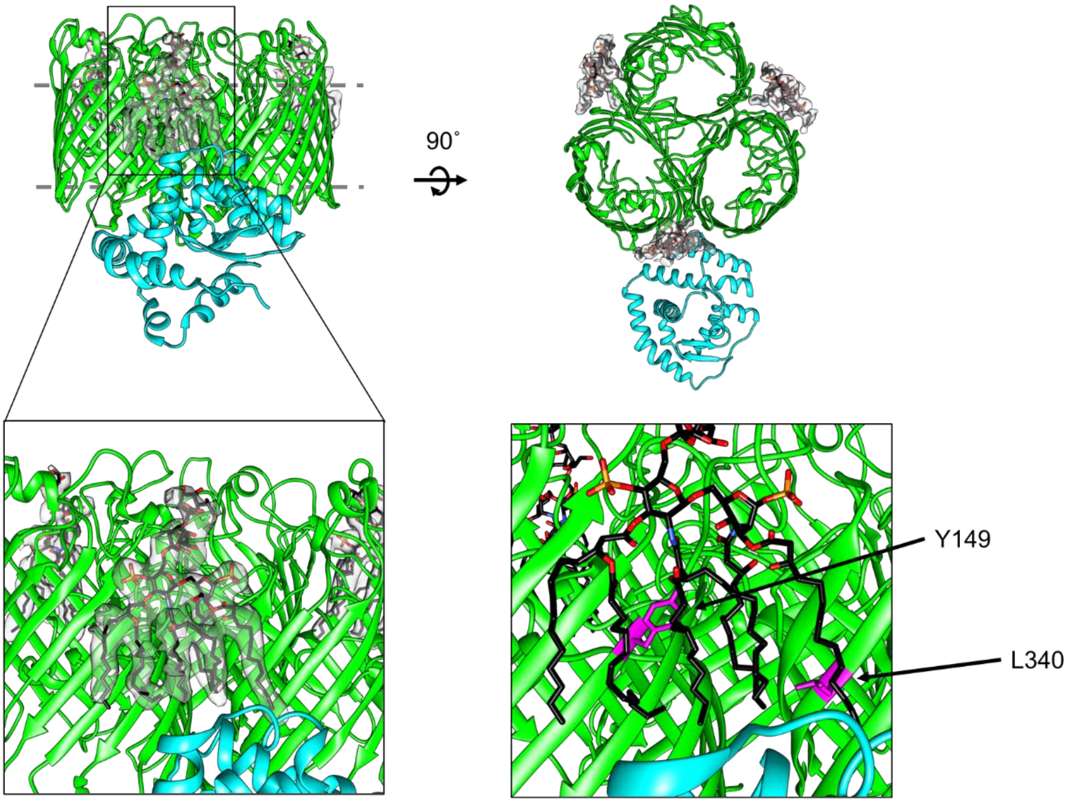

Supplementary Figure 13. LPS occupy interfaces of porin trimer subunits. Extra densities (*gray*; contour level 0.08) at porin trimer subunit interfaces in our $OmpC_3$-MlaA model (**PDB 8I8R**) can be confidently modelled as truncated LPS molecules (Kdo_2-Lipid A (KDL)). These LPS molecules occlude positions (Y149 and L340, *purple sticks*) on OmpC that enabled previously-reported photo-crosslinking to MlaA in the outer leaflet of the OM [3]. Cartoon illustrations are colored *green* (OmpC) and *cyan* (MlaA), respectively. Illustrations were generated using the software UCSF Chimera [2].

Supplementary Tables

Supplementary Table 1. Bacterial strains used in this study

Strains	Relevant genotypes and characteristics	References
MC4100	*F- araD139 Δ(argF-lac) U169 rpsL150 relA1 flbB5301 ptsF25 deoC1 ptsF25 thi*	[4]
NovaBlue	*endA1 hsdR17 (rK12– mK12+) supE44 thi-1 recA1 gyrA96 relA1 lac F′ proA+ B+ lacIq ZΔM15::Tn10*	Novagen
BL21(λDE3)	*fhuA2 lon ompT gal (λDE3) dcm ΔhsdS λDE3 = λ sBamHIo ΔEcoRI-B int::(lacI::PlacUV5::T7 gene1) i21 Δnin5*	Novagen
TKW001	BL21(λDE3) Δ*ompF*::*kan*	[3]
CZS010	MC4100 Δ*mlaA*::*kan*	[5]
YJ001	MC4100 Δ*mlaA*::*FRT* Δ*mlaC*::*kan*	This study
WY001	MC4100 Δ*mlaA::FRT* Δ*mlaD*::*kan*	[6]
CZS015	MC4100 Δ*ompC*::*kan*	[5]
YJ002	MC4100 Δ*ompC::FRT* Δ*mlaC*::*kan*	This study
CZS608	MC4100 Δ*ompC*::*ompC*$_{R92A}$; chromosomal *ompC* mutation introduced via a positive-negative selection	[3, 7]
CZS017	MC4100 Δ*pldA::kan*	[5]
CZS021	MC4100 Δ*pldA::FRT* Δ*mlaA::kan*	[5]
CZS219	MC4100 Δ*mlaC::FRT* Δ*pldA::kan*	[5]
RS112	MC4100 Δ*tolB*::*kan*	[8]
RS123	MC4100 Δ*pal*::*kan*	[8]

Supplementary Table 2. Plasmids used in this study

Plasmids	Relevant genotypes and characteristics	References
pET22/42	pT7(*lac*$_O$) inducible expression vector, contains multiple cloning site of pET42a(+) in pET22b(+) backbone; Amp^R	[9]
pET23/42	pT7 inducible expression vector, contains multiple cloning site of pET42a(+) in pET23a(+) backbone; Amp^R	[9]
pSup-BpaRS-6TRN	Encodes an orthogonal tRNA and aminoacyl-tRNA synthetase permitting ribosomal incorporation of *p*Bpa at TAG stop codons (pSup)	[10]
pCDFDuet-1	pT7 inducible expression vector; Spec^R	Novagen
pDSW206	Promoter down mutations in -35 and -10 of pTrc99a; Amp^R	[11]
pET23/42*mlaA-His*	Encodes full length MlaA with C-terminal His8 tag; Amp^R	[5]
pET23/42*mlaA*$_{3G3P}$-*His*	Encodes full length MlaA$_{3G3P}$ with C-terminal His8 tag; Amp^R	[3]
pET23/42*mlaA**-*His*	Encodes full length MlaA* with C-terminal His8 tag; Amp^R	[3]
pCDF*mlaA-His*	Encodes full length MlaA with C-terminal His8 tag; Spec^R	[3]
pCDF*mlaC*^{V171C}-*His-mlaA*^{Q205C}	Encodes full length MlaC^{V171C} with C-terminal His6 tag and full length MlaA^{Q205C}; Spec^R	This study
pET23/42*mlaC-mlaB*	Encodes full length MlaC and MlaB; Amp^R	Feifan Zhu
pET22/42*mlaC-His*	Encodes full length MlaC with C-terminal His8 tag; Amp^R	[6]
pDSW206*ompC*	Encodes full length OmpC; Amp^R	[3]
pDSW206*ompF*	Encodes full length OmpF; Amp^R	[3]
pDSW206*ompC-His*	Encodes full length OmpC with C-terminal His8 tag; Amp^R	[3]
pTrc99a-*pldA*	Encodes full length PldA; Amp^R	[5]

Supplementary Table 3. Primers used in this study.

Primers	Sequence (5' to 3')*
mlaA_R28B FP	GATCAGCAAGGG tag TCTGACCCGTTAGAAGGGTTC
mlaA_R28B RP	CTAACGGGTCAGA cta CCCTTGCTGATCTGTACCGG
mlaA_N41B FP	GCACCATGTAC tag TTCAACTTCAATGTATTAG
mlaA_N41B RP	TTGAAGTTGAA cta GTACATGGTGCGGTTGAAC
mlaA_N45B FP	ACTTCAACTTC tag GTATTAGACCCGTATATTG
mlaA_N45B RP	GGGTCTAATAC cta GAAGTTGAAGTTGTACATG
mlaA_P49B FP	AATGTATTAGAC tag TATATTGTTCGACCGGTC
mlaA_P49B RP	CGAACAATATA cta GTCTAATACATTGAAGTTG
mlaA_Y50B FP	TATTAGACCCG tag ATTGTTCGACCGGTCGCTG
mlaA_Y50B RP	GGTCGAACAAT cta CGGGTCTAATACATTGAAG
mlaA_P54B FP	TATATTGTTCGA tag GTCGCTGTCGCCTGGCG
mlaA_P54B RP	GGCGACAGCGAC cta TCGAACAATATACGGGTC
mlaA_A58B FP	CCGGTCGCTGTC tag TGGCGTGATTATGTTCCG
mlaA_A58B RP	ATAATCACGCCA cta GACAGCGACCGGTCGAAC
mlaA_D61B FP	GTCGCCTGGCGT tag TATGTTCCGCAACCGGCG
mlaA_D61B RP	TTGCGGAACATA cta ACGCCAGGCGACAGCGAC
mlaA_S181B FP	CTGGCCGATG tag GTGGGTAAATGGACGCTTG
mlaA_S181B RP	CATTTACCCAC cta CATCGGCCAGGTCAGCCAG
mlaA_V182B FP	GGCCGATGTCT tag GGTAAATGGACGCTTGAAG
mlaA_V182B RP	GTCCATTTACC cta AGACATCGGCCAGGTCAG
mlaA_G183B FP	GCCGATGTCTGTG tag AAATGGACGCTTGAAGGG
mlaA_G183B RP	CAAGCGTCCATTT cta CACAGACATCGGCCAGGTC
mlaA_T186B FP	GTGGGTAAATGG tag CTTGAAGGGATCGAAACCC
mlaA_T186B RP	GATCCCTTCAAG cta CCATTTACCCACAGACATCGG
mlaA_L187B FP	GGTAAATGGACG tag GAAGGGATCGAAACCCGCG
mlaA_L187B RP	GTTTCGATCCCTTC cta CGTCCATTTACCCACAGAC
mlaA_E188B FP	AAATGGACGCTT tag GGGATCGAAACCCGCG
mlaA_E188B RP	GTTTCGATCCC cta AAGCGTCCATTTACCCACAG
mlaA_G189B FP	TGGACGCTTGAA tag ATCGAAACCCGCGCTCAG
mlaA_G189B RP	GCGGGTTTCGAT cta TTCAAGCGTCCATTTACCC
mlaA_A194B FP	GATCGAAACCCGC tag CAGCTGCTGGATTCCGATG
mlaA_A194B RP	ATCCAGCAGCTG cta GCGGGTTTCGATCCCTTCAAG
mlaA_Q195B FP	GAAACCCGCGCT tag CTGCTGGATTCCGATGG
mlaA_Q195B RP	GAATCCAGCAG cta AGCGCGGGTTTCGATCCC
mlaA_D200B FP	CTCAGCTGCTGGATTCC tag GGTCTGCTGCG
mlaA_D200B RP	CGACTGACGCAGCAGACC cta GGAATCCAGCAG
mlaA_G201B FP	CTGGATTCCGAT tag CTGCTGCGTCAGTCGTCCG
mlaA_G201B RP	CTGACGCAGCAG cta ATCGGAATCCAGCAGCTG
mlaA_Q205B FP	CCGATGGTCTGCTGCGT tag TCGTCCGATCC
mlaA_Q205B RP	CATAATATAAGGATCGGACGA cta ACGCAGCAGAC
mlaA_D208B FP	CGTCAGTCGTCC tag CCTTATATTATGGTGCGC
mlaA_D208B RP	CATAATATAAGG cta GGACGACTGACGCAGCAG
mlaA_M212B FP	GATCCTTATATT tag GTGCGCGAAGCGTACTTC
mlaA_M212B RP	CGCTTCGCGCAC cta AATATAAGGATCGGACGAC
mlaA_E215B FP	TTATGGTGCGC tag GCGTACTTCCAGCGTCATG
mlaA_E215B RP	CTGGAAGTACGC cta GCGCACCATAATATAAGG
mlaA_A216B FP	ATGGTGCGCGAA tag TACTTCCAGCGTCATGAT
mlaA_A216B RP	ACGCTGGAAGTA cta TTCGCGCACCATAATATA

mlaA_Q219B FP	CGAAGCGTACTTC tag CGTCATGATTTCATCGC
mlaA_Q219B RP	AAATCATGACG cta GAAGTACGCTTCGCGCACC
mlaA_D222B FP	TTCCAGCGTCAT tag TTCATCGCTAATGGCGGC
mlaA_D222B RP	ATTAGCGATGAA cta ATGACGCTGGAAGTACGC
mlaA_F223B FP	AGCGTCATGAT tag ATCGCTAATGGCGGCGAAC
mlaA_F223B RP	CCATTAGCGAT cta ATCATGACGCTGGAAGTAC
mlaA_Q195C FP	GAAACCCGCGCT tgc CTGCTGGATTCCGATGG
mlaA_Q195C RP	GAATCCAGCAG gca AGCGCGGGTTTCGATCCC
mlaA_Q205C FP	CCGATGGTCTGCTGCGT tgc TCGTCCGATCC
mlaA_Q205C RP	CATAATATAAGGATCGGACGA gca ACGCAGCAGAC
mlaA_M212C FP	GATCCTTATATT tgc GTGCGCGAAGCGTACTTC
mlaA_M212C RP	CGCTTCGCGCAC gca AATATAAGGATCGGACGA
mlaA_F223C FP	AGCGTCATGAT tgc ATCGCTAATGGCGGCGAAC
mlaA_F223C RP	CCATTAGCGAT gca ATCATGACGCTGGAAGTAC
mlaC_V171C_FP	CATGATTGCTGAAGGC tgc AGTATGATCACCAC
mlaC_V171C_RP	ATCATACT gca GCCTTCAGCAATCATGTCGTAA
mlaC_K128C_FP	CCGCTGGGCGAT tgc ACCATTGTGCCTATTCGC
mlaC_K128C_RP	AGGCACAATGGT gca ATCGCCCAGCGGCTGTTC
mlaC_P132C_FP	AAACCATTGTG tgc ATTCGCGTTACCATTATTG
mlaC_P132C_RP	TGGTAACGCGAAT gca CACAATGGTTTTATCGC
mlaC_Q151C_FP	CGTCTGGACTTC tgc TGGCGTAAAAACTCCCAG
mlaC_Q151C_RP	GTTTTTACGCCA gca GAAGTCCAGACGCACCGG
mlaC_S172C_FP	TTGCTGAAGGCGTC tgc ATGATCACCACCAAAC
mlaC_S172C_RP	GTGGTGATCAT gca GACGCCTTCAGCAATCATG
mlaC_T175C_FP	GTCAGTATGATC tgc ACCAAACAAAACGAGTGG
mlaC_T175C_RP	GTTTTGTTTGGT gca GATCATACTGACGCCTTC
mlaA_CTD4 FP	GGCGAACTCAAA taa CAGGAAAATCCGAACGC
mlaA_CTD4 RP	GGATTTTCCTG tta TTTGAGTTCGCCGCCATT
mlaA_CTD5 FP	CAGGAAAATCCG taa GCACAAGCGATTCAGGATG
mlaA_CTD5 RP	GAATCGCTTGTGC tta CGGATTTTCCTGCGGTTTG
mlaA_CTD6 FP	CAAGCGATTCAG taa GATTTAAAAGATATTG
mlaA_CTD6 RP	CTTTTAAATC tta CTGAATCGCTTGTGCGTTC
mlaA_2DD2R FP	CAAGCGATTCAG cgc cgc TTAAAAGATATTGATTC
mlaA_2DD2R RP	ATATCTTTTAA gcg gcg CTGAATCGCTTGTGCGTTC
mlaA_2DE2R FP	GATTTAAAA cgc ATTGATTCT cgc CTCGAGCACCACC
mlaA_2DE2R RP	GCTCGAG gcg AGAATCAAT gcg TTTTAAATCATCCTG
mlaC0 FP	CGTACAGGTG gac TACGCCGGTGCGCTG
mlaC0 RP	ACCGGCGTA gtc CACCTGTACGTATGGC
mlaC1 FP	GTATTAC gac AGTGCGACCCCTGCTCAA gac GAAGCCTACT
mlaC1 RP	GCTTC gtc TTGAGCAGGGGTCGCACT gtc GTAATACTGGCC
mlaC2 FP	CCGCTTTC gac GAGTACCTG gac CAGGCTTACGGTCAG
mlaC2 RP	CGTAAGCCTG gtc CAGGTACTC gtc GAAAGCGGCAAAG
mlaC3 FP	ATTGACCCGAATGGC gac CCGCCGGTG gac CTGGACTTCC
mlaC3 RP	CCACTGGAAGTCCAG gtc CACCGGCGG gtc GCCATTCGGG
mlaC4 FP	CTTCCAGTGG gac gac AACTCCCAGACGGGCAAT
mlaC4 RP	CGTCTGGGAGTT gtc gtc CCACTGGAAGTCCAG
mlaC5 FP	GGAACGCTGCTG gac ACC gac GGTATCGACGGCCTG
mlaC5 RP	CGTCGATACC gtc GGT gtc CAGCAGCGTTCCCCACTC
mlaC7 FP	AACTG gac TCGATTTCTCAACAG gac ATCACTCTGGAAG
mlaC7 RP	GAGTGAT gtc CTGTTGAGAAATCGA gtc CAGTTGCGCAG
mlaA_M165C FP	GACGGTGGTGAT tgc GCGGATGGTTTTTACCCGG

mlaA_M165C RP	AAAACCATCCGC gca ATCACCACCGTCATCACGC
mlaA_A166C FP	GGTGGTGATATG tgc GATGGTTTTTACCCGGTTC
mlaA_A166C RP	GTAAAAACCATC gca CATATCACCACCGTCATC
mlaA_D167C FP	GACGGTGGTGATATGGCG tgc GGTTTTTACCCG
mlaA_D167C RP	AAGAACCGGGTAAAAACC gca CGCCATATCACC
mlaA_Y170C FP	GCGGATGGTTTT tgc CCGGTTCTTTCCTGGCTG
mlaA_Y170C RP	GGAAAGAACCGG gca AAAACCATCCGCCATATC
mlaA_V172C FP	GGTTTTTACCCG tgc CTTTCCTGGCTGACCTGG
mlaA_V172C RP	CAGCCAGGAAAG gca CGGGTAAAAACCATCCGC
mlaA_L173C FP	GATGGTTTTTACCCGGTT tgc TCCTGGCTGACC
mlaA_L173C RP	CGGCCAGGTCAGCCAGGA gca AACCGGGTAAAA
mlaA_S174C FP	TACCCGGTTCTT tgc TGGCTGACCTGGCCGATG
mlaA_S174C RP	CCAGGTCAGCCA gca AAGAACCGGGTAAAAACC
mlaA_W175C FP	TTTTACCCGGTTCTTTCC tgc CTGACCTGGCCG
mlaA_W175C RP	CAGACATCGGCCAGGTCAG gca GGAAAGAACCGG
mlaA_L176C FP	CTTTCCTGG tgc ACCTGGCCGATGTCTGTG
mlaA_L176C RP	CATCGGCCAGGT gca CCAGGAAAGAACCGGG
mlaA_T177C FP	CTTTCCTGGCTG tgc TGGCCGATGTCTGTGGG
mlaA_T177C RP	GACATCGGCCA gca CAGCCAGGAAAGAACCGG
mlaA_W178C FP	GTTCTTTCCTGGCTGACC tgc CCGATGTCTGTG
mlaA_W178C RP	ATTTACCCACAGACATCGG gca GGTCAGCCAGG
mlaA_P179C FP	GGCTGACCTGG tgc ATGTCTGTGGGTAAATGG
mlaA_P179C RP	CCCACAGACAT gca CCAGGTCAGCCAGGAAAG
mlaA_M180C FP	CTGACCTGGCCG tgc TCTGTGGGTAAATGGACG
mlaA_M180C RP	TTTACCCACAGA gca CGGCCAGGTCAGCCAGG
mlaA_S181C FP	CTGGCCGATG tgc GTGGGTAAATGGACGCTTG
mlaA_S181C RP	CATTTACCCAC gca CATCGGCCAGGTCAGCCAG
mlaA_W185C FP	CTGTGGGTAAA tgc ACGCTTGAAGGGATCGAAAC
mlaA_W185C RP	CCCTTCAAGCGT gca TTTACCCACAGACATCG
mlaA_L187C FP	GGTAAATGGACG tgc GAAGGGATCGAAACCCGCG
mlaA_L187C RP	GTTTCGATCCCTTC gca CGTCCATTTACCCACAGAC
mlaA_E188C FP	AAATGGACGCTT tgc GGGATCGAAACCCGCGC
mlaA_E188C RP	GTTTCGATCCC gca AAGCGTCCATTTACCCAC
mlaA_R193C FP	CTTGAAGGGATCGAAACC tgc GCTCAGCTGCTG
mlaA_R193C RP	GGAATCCAGCAGCTGAGC gca GGTTTCGATCCC

Supplementary Table 4. List of all *p*Bpa crosslinking sites tested on MlaA.

Ec (*Kp*) Residues	Crosslink to MlaC
R28	-
N41	-
N45	-
P49	-
Y50	Weak
P54	-
A58	Weak
D61	-
D92	-
S181	-
V182 (I182)	-
G183	-
T186 (A186)	-
L187 (V187)	-
E188	-
G189	-
A194	Weak
Q195	Strong
D200	-
G201	Strong
Q205	Strong
S206	Moderate
D208	Moderate
M212 (V212)	Strong
E215	Moderate
A216	Moderate
Q219	Moderate
D222	-
F223	Strong

Supplementary Table 5. Summary of cryo-EM data collection and model refinement.

	OmpC$_3$-(MlaA-MlaC)$_{1-3}$ (EMDB-35250) (PDB 8I8R)	OmpC$_3$-(MlaA-MlaC)$_3$ (EMDB-35251)	OmpC$_3$-(MlaA-MlaC)$_2$ (EMDB-35252)	OmpC$_3$-(MlaA-MlaC) (EMDB-35253) (PDB 8I8X)
**Data collection and processing**				
Magnification	105,000x	105,000x	105,000x	105,000x
Voltage (kV)	300	300	300	300
Electron exposure (e–/Å^2)	90	90	90	90
Defocus range (μm)	-0.8 to -2.0	-0.8 to -2.0	-0.8 to -2.0	-0.8 to -2.0
Pixel size (Å)	0.834	0.834	0.834	0.834
Symmetry imposed	C1	C1	C1	C1
Initial particle images (no.)	2,434,319	-	-	-
Final particle images (no.)	205,487	38,528	90,496	76,463
Map resolution (Å) FSC threshold	2.93 (0.143)	3.42 (0.143)	3.19 (0.143)	3.25 (0.143)
Map resolution range (Å)	2.4-3.6	-	-	2.2-7.0
**Refinement**	**OmpC-MlaA**			**OmpC-MlaA-MlaC**
Initial model used (PDB code)	5NUP			5NUP
Model resolution (Å) FSC threshold	-	-	-	-
Model resolution range (Å)	-	-	-	-
Map sharpening *B* factor (Å^2)	88.0	50.2	73.8	69.1
**Composition**				
Chains	7			8
Protein residues	1,232			1,450
Non-hydrogen atoms	10,151			11,226
Ligands	3			3
R.m.s. deviations				
Bond lengths (Å)	0.002			0.007
Bond angles (°)	0.622			1.174
Validation				
MolProbity score	1.73			1.92
Clashscore	4.14			4.40
Poor rotamers (%)	1.82			0.71
Ramachandran plot				
Favored (%)	0.16			0.28
Allowed (%)	7.11			7.71
Disallowed (%)	92.73			92.01

Supplementary References

1. Punjani A, Rubinstein JL, Fleet DJ, Brubaker MA. cryoSPARC: algorithms for rapid unsupervised cryo-EM structure determination. *Nat Methods* **14**, 290-296 (2017).

2. Pettersen EF, *et al.* UCSF Chimera--a visualization system for exploratory research and analysis. *J Comput Chem* **25**, 1605-1612 (2004).

3. Yeow J, *et al.* The architecture of the OmpC-MlaA complex sheds light on the maintenance of outer membrane lipid asymmetry in Escherichia coli. *J Biol Chem* **293**, 11325-11340 (2018).

4. Casadaban MJ. Transposition and fusion of the lac genes to selected promoters in Escherichia coli using bacteriophage lambda and Mu. *J Mol Biol* **104**, 541-555 (1976).

5. Chong ZS, Woo WF, Chng SS. Osmoporin OmpC forms a complex with MlaA to maintain outer membrane lipid asymmetry in Escherichia coli. *Mol Microbiol* **98**, 1133-1146 (2015).

6. Ercan B, Low WY, Liu X, Chng SS. Characterization of Interactions and Phospholipid Transfer between Substrate Binding Proteins of the OmpC-Mla System. *Biochemistry* **58**, 114-119 (2019).

7. Khetrapal V, Mehershahi K, Rafee S, Chen S, Lim CL, Chen SL. A set of powerful negative selection systems for unmodified Enterobacteriaceae. *Nucleic Acids Res* **43**, e83 (2015).

8. Shrivastava R, Jiang X, Chng SS. Outer membrane lipid homeostasis via retrograde phospholipid transport in Escherichia coli. *Mol Microbiol* **106**, 395-408 (2017).

9. Wu T, McCandlish AC, Gronenberg LS, Chng SS, Silhavy TJ, Kahne D. Identification of a protein complex that assembles lipopolysaccharide in the outer membrane of Escherichia coli. *Proc Natl Acad Sci U S A* **103**, 11754-11759 (2006).

10. Ryu Y, Schultz PG. Efficient incorporation of unnatural amino acids into proteins in Escherichia coli. *Nat Methods* **3**, 263-265 (2006).

11. Weiss DS, Chen JC, Ghigo JM, Boyd D, Beckwith J. Localization of FtsI (PBP3) to the septal ring requires its membrane anchor, the Z ring, FtsA, FtsQ, and FtsL. *J Bacteriol* **181**, 508-520 (1999).
