# Supplementary material for: Molecular mechanism of phospholipid transport at the bacterial outer membrane interface
Source: Nat Commun. 2023 Dec 13;14:8285. doi: 10.1038/s41467-023-44144-8 (PMC10719372; doi:10.1038/s41467-023-44144-8)
Supplement: Supplementary file 3 — Reporting Summary [file 41467_2023_44144_MOESM3_ESM.pdf]

nature portfolio

Corresponding author(s): Shu-Sin Chng

Last updated by author(s): Nov 28, 2023

Reporting Summary

Nature Portfolio wishes to improve the reproducibility of the work that we publish. This form provides structure for consistency and transparency in reporting. For further information on Nature Portfolio policies, see our Editorial Policies and the Editorial Policy Checklist.

Statistics

For all statistical analyses, confirm that the following items are present in the figure legend, table legend, main text, or Methods section.

n/a	Confirmed	
☐	☒	The exact sample size (n) for each experimental group/condition, given as a discrete number and unit of measurement
☒	☐	A statement on whether measurements were taken from distinct samples or whether the same sample was measured repeatedly
☒	☐	The statistical test(s) used AND whether they are one- or two-sided<br>*Only common tests should be described solely by name; describe more complex techniques in the Methods section.*
☒	☐	A description of all covariates tested
☒	☐	A description of any assumptions or corrections, such as tests of normality and adjustment for multiple comparisons
☐	☒	A full description of the statistical parameters including central tendency (e.g. means) or other basic estimates (e.g. regression coefficient) AND variation (e.g. standard deviation) or associated estimates of uncertainty (e.g. confidence intervals)
☒	☐	For null hypothesis testing, the test statistic (e.g. F, t, r) with confidence intervals, effect sizes, degrees of freedom and P value noted<br>*Give P values as exact values whenever suitable.*
☒	☐	For Bayesian analysis, information on the choice of priors and Markov chain Monte Carlo settings
☒	☐	For hierarchical and complex designs, identification of the appropriate level for tests and full reporting of outcomes
☒	☐	Estimates of effect sizes (e.g. Cohen's d, Pearson's r), indicating how they were calculated

Our web collection on statistics for biologists contains articles on many of the points above.

Software and code

Policy information about availability of computer code

Data collection	All cryo-EM movies were recorded in counting mode with SerialEM4 (Mastronarde, 2005).
Data analysis	cryoSPARC 4.0 (Punjani et al., 2017) was used to process the EM data. Initial atomic models were built using SWISS-MODEL (Waterhouse et al., 2018), (Pettersen et al., 2004), followed by density-fitting in COOT 0.9.8 (Emsley & Cowtan, 2004). Refinement in RealSpace using the program of Phenix 1.20.1-4487 (Liebschner et al., 2019), final coordinates of the asymmetric units were checked using MolProbity (Chen et al., 2010).

For manuscripts utilizing custom algorithms or software that are central to the research but not yet described in published literature, software must be made available to editors and reviewers. We strongly encourage code deposition in a community repository (e.g. GitHub). See the Nature Portfolio guidelines for submitting code & software for further information.

Data

Policy information about availability of data

All manuscripts must include a data availability statement. This statement should provide the following information, where applicable:

- Accession codes, unique identifiers, or web links for publicly available datasets
- A description of any restrictions on data availability
- For clinical datasets or third party data, please ensure that the statement adheres to our policy

Four 3D cryo-EM maps of OmpC3-(MlaA-MlaC) have been deposited in the Electron Microscopy Data Bank under accession numbers EMD-35250 (OmpC3-(MlaA-MlaC)1-3), EMD-35251 (OmpC3-(MlaA-MlaC)3), EMD-35252 (OmpC3-(MlaA-MlaC)2), EMD-35253 (OmpC3-(MlaA-MlaC)). Two atomic coordinate files have also

been deposited in the Protein Data Bank under the accession numbers 8I8R (OmpC3-MlaA) and 8I8X (OmpC3-MlaA-MlaC). Accessibility has been designated as "for immediate release on publication" (HPUB).

Research involving human participants, their data, or biological material

Policy information about studies with human participants or human data. See also policy information about sex, gender (identity/presentation), and sexual orientation and race, ethnicity and racism.

Reporting on sex and gender	This information has not been collected.
Reporting on race, ethnicity, or other socially relevant groupings	This information has not been collected.
Population characteristics	This information has not been collected.
Recruitment	This information has not been collected.
Ethics oversight	This information has not been collected.

Note that full information on the approval of the study protocol must also be provided in the manuscript.

Field-specific reporting

Please select the one below that is the best fit for your research. If you are not sure, read the appropriate sections before making your selection.

[x] Life sciences [ ] Behavioural & social sciences [ ] Ecological, evolutionary & environmental sciences

For a reference copy of the document with all sections, see nature.com/documents/nr-reporting-summary-flat.pdf

Life sciences study design

All studies must disclose on these points even when the disclosure is negative.

Sample size	Biological triplicates of immunoblots were visualised, and yielded the same data (n=3).
Data exclusions	No data were excluded from the analyses.
Replication	Biological triplicates (n=3) were performed and a representative immunoblot was used. All attempts at replication were successful.
Randomization	Randomization was not possible for these in vitro experiments.
Blinding	Blinding was not possible for these in vitro experiments.

Reporting for specific materials, systems and methods

We require information from authors about some types of materials, experimental systems and methods used in many studies. Here, indicate whether each material, system or method listed is relevant to your study. If you are not sure if a list item applies to your research, read the appropriate section before selecting a response.

Materials & experimental systems

n/a	Involved in the study	
[ ]	[x]	Antibodies
[x]	[ ]	Eukaryotic cell lines
[x]	[ ]	Palaeontology and archaeology
[x]	[ ]	Animals and other organisms
[x]	[ ]	Clinical data
[x]	[ ]	Dual use research of concern
[x]	[ ]	Plants

Methods

n/a	Involved in the study	
[x]	[ ]	ChIP-seq
[x]	[ ]	Flow cytometry
[x]	[ ]	MRI-based neuroimaging

Antibodies

Antibodies used	Penta®His HRP Conjugate (Qiagen, Lot No.: 175023485) was used at a dilution of 1:5,000.<br>Mouse α-MlaA primary antibody (Abmart, Production ID: 19421-1-3/C385) was used at a dilution of 1:3000.<br>Secondary ECL™ Anti-Mouse IgG, HRP linked whole antibody (from sheep) (GE Healthcare, Lot No.: 17170583) was used at a dilution of 1:3000.

	Rabbit α-MlaC primary antibody was used at a dilution of 1:500 (Pacific Immunology, Production ID.: 11357).<br>Secondary ECL™ Anti-Rabbit IgG, HRP linked whole antibody (from donkey) (GE Healthcare, Lot.: 17197685) was used at a dilution of 1:3000.
Validation	Monoclonal α-MlaA antibody (Abmart, Production ID: 19421-1-3/C385) was developed against the MlaA VGKWTLEGIETRAQ peptide sequence (a.a. 182-195 of MlaA) and used at 1:3,000 dilution. Immunization of mice was performed by Abmart (China).
